# Using the Acute Flaccid Paralysis Surveillance System to Identify Cases of Acute Flaccid Myelitis, Australia, 2000‒2018

**DOI:** 10.3201/eid2801.211690

**Published:** 2022-01

**Authors:** Liz J. Walker, Bruce R. Thorley, Anne Morris, Elizabeth J. Elliott, Nathan Saul, Philip N. Britton, on behalf of the Australian Polio Expert Panel

**Affiliations:** The Australian National University, Canberra, Australian Capital Territory, Australia (L.J. Walker);; Australian Government Department of Health, Canberra (L.J. Walker, N. Saul);; Peter Doherty Institute for Infection and Immunity, Melbourne, Victoria, Australia (B.R. Thorley);; The Children’s Hospital at Westmead, Sydney, New South Wales, Australia (A. Morris, E.J. Elliott, P.N. Britton);; The University of Sydney, Sydney (E.J. Elliott, P.N. Britton)

**Keywords:** acute flaccid myelitis, central nervous system infections, enterovirus infections, enteroviruses, viruses, poliomyelitis, enterovirus A, enterovirus D, humans, acute flaccid paralysis surveillance system, Australia

## Abstract

Since 2012, the United States has reported a distinct syndrome of acute flaccid paralysis (AFP) with anterior myelitis, predominantly in children. This polio-like syndrome was termed acute flaccid myelitis (AFM). Australia routinely conducts AFP surveillance to exclude poliomyelitis. We reviewed 915 AFP cases in Australia for children <15 years of age during 2000‒2018 and reclassified a subset to AFM by using the US Council of State and Territorial Epidemiologists case definition. We confirmed 37 AFM cases by using magnetic resonance imaging findings and 4 probable AFM cases on the basis of cerebrospinal fluid pleocytosis. Nonpolio enteroviruses were detected in 33% of AFM cases from which stool samples were tested. Average annual AFM incidence was 0.07 cases/100,000 person-years in children <15 years of age. AFM occurred sporadically in Australia before 2010 but regularly since then, indicating sustained, albeit rare, clinical manifestation in children. The AFP surveillance system in Australia is well-positioned to identify future AFM cases.

## Abstract 2

This system is well-positioned to rapidly identify future cases of acute flaccid myelitis.

During 2012 and 2014, reports of a distinct syndrome of acute flaccid paralysis (AFP) with inflammation of the spinal cord restricted predominately to the gray matter occurred in children in the United States (*1*,*2*). Clinicians referred to these patients as having a polio-like syndrome because stool samples were negative for polioviruses. To avoid confusion with poliomyelitis, clinicians called the syndrome acute flaccid myelitis (AFM). During August 2014, the US Centers for Disease Control and Prevention (CDC) put out a national call for cases of AFM and, during 2015, began passive surveillance for the condition under a standardized case definition (*3*). By August 2021, a total of 665 cases were reported (*4*). Outbreaks occurred biennially in late summer to autumn during 2014 (120 cases), 2016 (153 cases), and 2018 (238 cases) (*5*). A smaller number of AFM cases were reported from Europe, Asia, South America, Africa, and Oceania (*6*). The anticipated biennial outbreak in 2020 did not materialize, probably affected by coronavirus disease restrictions, such as mask wearing and physical distancing.

Children who have AFM show acute flaccid limb weakness, typically with asymmetric onset, affecting the arms more than the legs and proximal muscles more than distal muscles, with or without cranial nerve involvement (*7*). Patients frequently have an acute antecedent illness, most commonly respiratory. Analysis of cerebrospinal fluid (CSF) often shows an increased leukocyte count, supporting an infectious etiology. Although uncommon, AFM can be life-threatening. The health of affected patients can deteriorate quickly; >50% of children in the United States are admitted to an intensive care unit, and 1/4 of these children require mechanical ventilation (*3*). 

The standard for confirming a central nervous system viral infection is amplification of viral nucleic acid from CSF or detection of specific antibodies in the CSF (*8*,*9*). Detection of viral nucleic acid can be insensitive because the virus might have already been cleared from the CSF between the period of illness onset and the paralysis that prompts a lumbar puncture (*10*). The CDC detected nonpolio enteroviruses (NPEV) enterovirus D68 (EV-D68), enterovirus A71 (EV-A71), and coxsackievirus A16 in the CSF in a small number of patients, although NPEV were more often detected in specimens from other sites (*3*,*11*).

## AFP Surveillance

AFP surveillance began in Australia during 1995 by the Australian Paediatric Surveillance Unit (APSU) as part of Australia’s commitment to the global eradication of poliomyelitis (*12*). Every month, the APSU sends report cards to ≈1,500 pediatricians and other child health specialists in urban, rural, and remote regions, inquiring whether or not they have seen a newly diagnosed patient who has AFP and other selected conditions under surveillance (*13*). Since 2000, the monthly response rate has been >90%. In late 2007, the Paediatric Active Enhanced Disease Surveillance (PAEDS) network was established in tertiary hospitals to identify children hospitalized because of AFP and to complement the existing APSU surveillance. The aim of PAEDS was to help maintain the annual detection rate of AFP for Australia (the World Health Organization [WHO] target is at least 1 nonpolio AFP case/100,000 children <15 years of age/year), improve stool collection (the WHO target for a polio-free country is 2 stool samples within 14 days of symptom onset for >80% of nonpolio AFP cases), and assist in excluding poliovirus infection. PAEDS nursing staff identify AFP cases by actively screening hospital admissions at sentinel sites and matching hospital data with the AFP case definition. The APSU and PAEDS network have research ethics committee approval, and both operate under a waiver of consent.

Stool samples from AFP cases are sent to the National Enterovirus Reference Laboratory (NERL), which performs virus culture for the isolation of poliovirus and screens specimens for enterovirus RNA and reverse transcription PCR. The Polio Expert Panel (PEP) is convened 6 times a year to review and classify cases as poliomyelitis, polio-compatible, or the most likely clinical diagnosis for nonpolio AFP cases by using clinical and laboratory data and expert judgement.

In the past few decades, many high-income countries, including the United States and the United Kingdom, have failed to meet the annual WHO AFP target because of progressive decreases in reported cases. With the apparent emergence of AFM, the PEP recognized that the longstanding AFP surveillance system of Australia, in a high-income setting, afforded a unique opportunity to retrospectively analyze existing data and apply the new AFM case definition to identify cases or clusters of AFM that might have occurred in Australia. We describe the epidemiology, clinical, and diagnostic characteristics of these AFM cases.

## Methods

**Figure 1:**
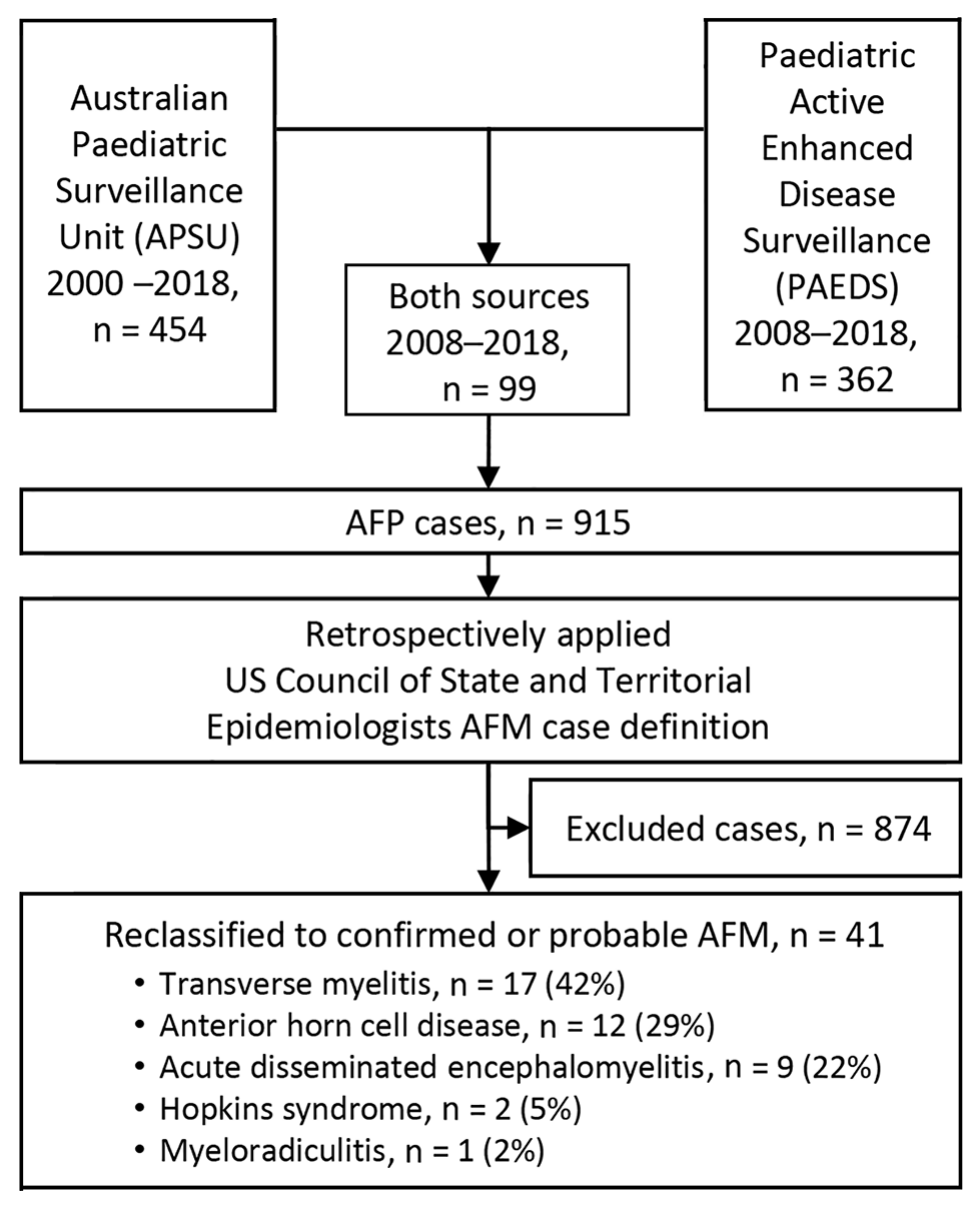
Flowchart of AFM reclassification by using the AFP surveillance system, Australia, 2000‒2018. AFM, acute flaccid myelitis; AFP, acute flaccid paralysis.

We reviewed 915 confirmed AFP cases reported to the AFP surveillance system from the APSU during 2000‒2018 and the PAEDS network during 2008‒2018 (Figure 1). Case reports included patient demographics, clinical features, diagnostic summary reports (magnetic resonance imaging [MRI], nerve conduction studies [NCS], and electromyograms [EMG]), and the final diagnosis assigned by the PEP. When duplicate reports were identified, questionnaires from all reporting pediatricians were consolidated into a single record for analysis. We identified cases of AFM by applying the US Council of State and Territorial Epidemiologists case definition to all AFP cases (*14*). A confirmed AFM case was required to have acute onset of flaccid limb weakness and confirmatory laboratory evidence by MRI showing a spinal cord lesion restricted largely to gray matter and spanning >1 spinal segments. A probable AFM case was required to have an acute onset of flaccid limb weakness and supportive laboratory evidence of CSF with pleocytosis (leukocyte count >5 cells/mm^3^).

The PEP, which includes neurologists, reviews all MRI reports for patients upon initially classification of cases as poliomyelitis, polio-compatible, or nonpolio AFP. For this analysis, we re-reviewed all MRI reports by using CDC definitions for AFM criteria. MRI spinal cord reports with terms such as affecting mostly gray matter, affecting the anterior horn or anterior horn cells, affecting the ventral horns, affecting the central cord, anterior myelitis, or poliomyelitis (with no supporting laboratory detection of polio) were all considered AFM-consistent terminology (i.e., a spinal cord lesion restricted largely to gray matter). Almost all MRIs were performed at children’s hospitals, and reporting was done by pediatric radiologists with the requisite expertise to delineate such changes. There were no cases of wild poliovirus‒associated poliomyelitis, and only 1 case involved a Sabin vaccine strain of poliovirus during the study period, which was excluded. Members of the PEP reviewed and confirmed cases as meeting AFM diagnostic criteria.

We used Stata 13 (StataCorp LLC, https://www.stata.com) for descriptive analyses and calculated annual AFM incidence rates by using population denominator data cubes from the Australian Bureau of Statistics (Australian Historical Population Statistics and Estimated Resident Population for 2017 and 2018). The study was approved by the Australian National University Human Research Ethics Committee (2019/472).

## Results

We identified 37 confirmed AFM cases on the basis of MRI findings and 4 probable AFM cases on the basis of pleocytosis in CSF. The original diagnoses of the 37 cases newly classified AFM cases included 17 (42%) cases of transverse myelitis, 12 (29%) cases of anterior horn cell disease, 9 (22%) cases of acute disseminated encephalomyelitis, 2 (5%) cases of Hopkins syndrome, and 1 (2%) case of myeloradiculitis. Cases of AFM accounted for 4% (41/915) of all confirmed AFP cases reported to the surveillance system in Australia. AFM was rare when compared with the most common diagnoses associated with AFP, which were Guillain–Barré syndrome (338 [37%] of 915), transverse myelitis (144 [16%] of 915), and acute disseminated encephalomyelitis (104 [11%] of 915).

AFM showed a near equal sex distribution, and the median age of onset was 4.07 (interquartile range 2.27–9.27) years (Table 1). A confirmed or probable prodromal illness was detected in 21 (51%) of 41 AFM cases. When a laboratory confirmation of an infection was reported, most (5/6, 83%) were upper respiratory tract infections. Bilateral lower limb paralysis was the most frequent site of weakness (13/39, 33%), followed by unilateral upper limb paralysis (11/39, 28%). A total of 34% (14/41) of AFM cases had bulbar or cranial nerve palsy. Sensation was typically intact (34/41, 83%); however, bladder or bowel dysfunction occurred in 41% (17/41). We found pleocytosis in 80% (33/41) of AFM cases. The median CSF leukocyte count was 38 leukocytes/mm^3^. An elevated protein level (>0.55 g/L) in CSF was observed for 20% (8/41) of cases.

**Table 1:** Characteristics of cases of acute flaccid myleitis for 41 children <15 years of age, Australia, 2000‒2018*

Characteristic	Value
Sex	
M	19 (46.3)
F	22 (53.7)
Median age, y, at onset (range)	4.07 (0.25–14.08)
Prodromal illness	
Confirmed	6 (14.6)
Probable	15 (36.6)
No	13 (31.7)
Unknown	7 (17.1)
Confirmed specified	
URTI	5 (83.3)
Gastrointestinal	1 (16.7)
Site of paralysis	
Known	39 (95.1)
Unknown	2 (4.9)
Site of paralysis, upper limbs	
Unilateral	11 (28.2)
Bilateral	3 (7.7)
Site of paralysis, lower limbs	
Unilateral	3 (7.7)
Bilateral	13 (33.3)
Site of paralysis, upper and lower limbs	
Unilateral	5 (12.8)
Bilateral	4 (10.3)
Bulbar/cranial nerve palsy	
Yes	14 (34.1)
No	26 (63.4)
Unknown	1 (2.4)
Reduced sensation	
Yes	6 (14.6)
No	34 (82.9)
Unknown	1 (2.4)
Bladder/bowel dysfunction	
Yes	17 (41.5)
No	15 (36.5)
Unknown	9 (22.0)
Median CSF protein level, g/L (range)	0.43 (0.11–1.18)
Elevated CSF protein level >0.55 g/L	
Yes	8 (19.5)
No	29 (70.7)
Unknown	4 (9.8)
Median CSF leukocyte count/mm^3^ (range)	38 (0–267)
Pleocytosis >5 cells mm^3^	
Yes	33 (80.5)
No	3 (7.3)
Unknown	5 (12.2)

*Values are no. (%) unless otherwise indicated. CSF, cerebrospinal fluid; URTI, upper respiratory tract infection.

MRI reports were available for 90% (37/41) of AFM cases, and all reports included terminology equivalent to a spinal cord lesion or lesions restricted mainly to the gray matter of the spine (Table 2). When the spinal region was specified, most (12/33, 36%) lesions were localized to the cervical spine, followed by longer lesions spanning the cervical-to-thoracic (7/33, 21%) and cervical-to-lumbar regions (7/33, 21%). An abnormal brain MRI was observed for 19% (7/37) of AFM cases. A total of 37% (11/37) of MRI reports (11/37) indicated abnormalities in the conus medullaris and spinal nerve roots of the cauda equina. NCS (14/41, 34%) and EMG (10/41, 24%) results were infrequently reported. Of the 14 cases in which NCS were performed, half of results were normal (7/14, 50%), 29% (4/14) were abnormal (including 2 case reports that did not describe the abnormality), and 21% (3/41) had no report. All abnormal NCS results indicated changes in motor amplitudes, suggesting active denervation or early recovery from mild denervation. A total of 60% (6/10) of EMG studies had abnormal results, 20% (2/10) had normal results, and 20% (2/10) had no report.

**Table 2:** Characteristics of cases of acute flaccid myelitis, by magnetic resonance imaging, for 41 children <15 years of age, Australia, 2000‒2018*

Characteristic	No. (%)
Magnetic resonance conducted	
Yes	37 (90.2)
No	4 (9.8)
Unknown	0
Brain abnormal	
Yes	7 (18.9)
No	30 (81.1)
Brain region	
White	1 (14.3)
Gray	1 (14.3)
Both	5 (71.4)
Brain stem abnormal	
Yes	5 (13.5)
No	32 (86.5)
Spinal cord abnormal	
Yes	33 (89.2)
Yes, no regional details	4 (10.8)
Spinal cord region abnormal	
Cervical	12 (36.4)
Thoracic	3 (9.1)
Lumbar	2 (6.1)
Cervical-thoracic	7 (21.2)
Thoracic-lumbar	2 (6.1)
Cervical-thoracic-lumbar	7 (21.2)
Restricted to gray matter	
Yes	37 (100.0)
No	0
Conus and roots abnormal	
Yes	11 (29.7)
No	26 (0.3)


**Figure 2:**
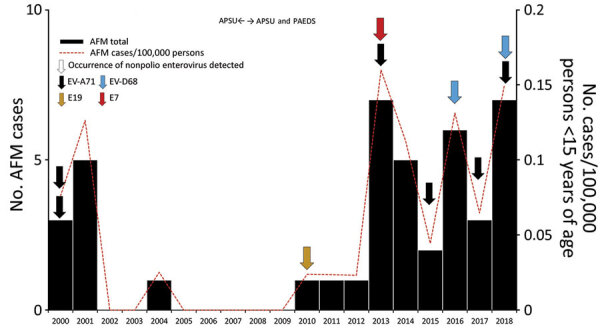
Annual number of cases of AFM and rate (per 100,000 persons) in 41 children <15 years of age and enteroviruses identified in stool specimens, Australia, 2000‒2018. Horizontal arrows indicate years when surveillance for AFM was conducted by the APSU and by both APSU and PAEDS. AFM, acute flaccid myelitis; APSU, Australian Paediatric Surveillance Unit; E, echovirus; EV, enterovirus; PAEDS, Paediatric Active Enhanced Disease Surveillance Network.

The annual frequency of AFM case counts ranged from 0 to 7 (Figure 2). We detected AFM during 2000–2001, 2004, and every year from 2010 on. Peaks in AFM occurred in 2001 (5 cases), 2013 (7 cases), 2016 (6 cases), and 2018 (7 cases). The average annual AFM incidence during 2000‒2007 was 0.03 cases/100,000 person-years in children <15 years of age. After inclusion of the PAEDS sites, we found that average annual AFM incidence during 2008‒2018 was 0.07 cases/100,000 person-years in children <15 years of age. The absence of cases during 2002–2003 and 2005‒2009 appeared genuine because of the sensitive nature of the AFP case definition required to identify poliomyelitis cases. AFM cases occurred in all Australia jurisdictions, except the Australian Capital Territory. No spatial clustering at the postal code level was identified.

**Figure 3:**
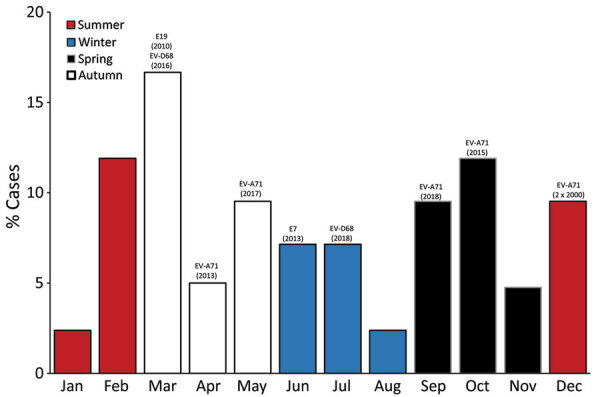
Temporal distribution of cases of acute flaccid myelitis, by month and seasonality, in 41 children <15 years of age, Australia, 2000‒2018. Text over bars indicates, where applicable, the associated enterovirus identified in a stool specimen and the year of onset of acute flaccid myelitis. E, echovirus; EV, enterovirus.

A total of 73% (30/41) of AFM cases had stool specimens sent to the NERL for enterovirus culture, reverse transcription PCR, and typing by sequencing a fragment of the viral protein 1 genomic region. Nonpolio enteroviruses were reported from 33% (10/30) of cases, including EV-A71 (6 cases, 20%), EV-D68 (2 cases, 7%), echovirus 7 (1 case, 3%), and echovirus 19 (1 case, 3%). All enteroviruses were detected in stool samples except for 1 EV-D68 case, which was identified in an additional respiratory specimen. The B4 and C4 subgenotypes of EV-A71 were detected in AFM cases during 2000 and 2013, respectively, as part of known EV-A71 outbreaks; and subgenotypes B5, C4, and C6 were detected sporadically in AFM cases during 2015, 2017 and 2018, respectively (Figure 2). Enterovirus-D68 subgenotype B3 was detected in an AFM case during 2016 and 2018. March (early autumn) was the most common month for AFM cases (7 cases), followed by February (late summer) and October (mid-spring) (5 cases each). (Figure 3).

## Discussion

Acute flaccid myelitis was described in the United States during 2012 (*3*). Countries in Europe, Asia and the Americas later detected cases. Using data from the AFP surveillance system in Australia, we identified that AFM occurred in Australia in the early 2000s and was associated with outbreaks of EV-A71 infection; and hand, foot, and mouth disease, and that sustained detection of AFM after 2010 was associated with EV-A71, EV-D68, echovirus 7, and echovirus 19.

After the PAEDS network was introduced to the AFP surveillance system, we calculated that the average incidence of AFM in children <15 years of age was 0.07 cases/100,000 person-years. The highest peak during 2013, which was 0.16 cases/100,000 person-years, coincided with an outbreak of EV-A71 in metropolitan Sydney (*15*). In a cohort study of children 1‒18 years of age in northern California, USA, the estimated incidence of AFM increased from 0.30 cases/100,000 person-years to 1.43 cases/100,000 person-years during 2011‒2016 (*16*). When Elrick et al. re-evaluated 45 cases in the United States that met the AFM case definition; they found that 11 cases (24%) had alternative diagnoses (e.g., transverse myelitis, spinal cord ischemia, other demyelinating syndromes, polyradiculoneuropathy, meningitis, and Chiari I malformation) (*17*). This finding suggests that incidence rates of AFM in the United States might be inflated by other conditions associated with acute paralysis.

We postulate that the lower incidence of AFM in Australia might occur because the AFP surveillance system reports all cases of AFP, not solely AFM, and that the PEP clinically reviews all cases and considers a range of differential diagnoses simultaneously. Although incidence rates based on small numbers should be interpreted with caution, we believe that presenting them enables a comparison of AFM incidence between countries and a baseline for future epidemiologic studies in Australia. The uptick in AFM detection from 2013 on supports the apparent increase in circulating, neuroinvasive, nonpolio enteroviruses internationally (*15*,*18*). However, the increase might also be caused by increased awareness about the AFM by pediatricians or increased availability of diagnostic MRI.

Identifying of the causative agent of AFM in CSF samples has proven elusive in most cases. However, the Bradford Hill criteria support a causal relationship between EV-D68 and AFM (*19*). The challenge to detect enterovirus RNA in CSF led Schubert et al. (*9*) to use pan-viral serologic analysis. They assessed the CSF from children with AFM against other pediatric neurologic controls for intrathecal antiviral antibodies. They found that 69% of AFM cases had elevated levels of antibodies against enteroviruses compared with 7% for controls (*9*). In the absence of direct detection of a pathogen by using molecular techniques, pan-viral serologic analysis might provide evidence that nonpolio enteroviruses play a causal role in AFM.

During 2014, a nationwide outbreak of EV-D68 respiratory disease was associated with a large outbreak of AFM in the United States (*20*). In Australia, AFP surveillance detected EV-D68 during 2008, and peaks of activity occurred during 2011 and 2013 in laboratory-based enterovirus surveillance (*21*). EV-D68 was detected in 1 stool specimen associated with an AFM case in July 2018, and a review of PAEDS data for 2007‒2017 found EV-D68 in a respiratory specimen of another AFM case during March 2016 (*22*). Both viruses were identified as EV-D68 subgenotype B3, which was detected in increasing numbers in Europe and the United States during 2016 (*23**,**24*).

The NERL reported 4 other detections of EV-D68 in stool specimens from AFP cases during 2010 (1 case, genotype A), 2016 (1 case, subgenotype B3), and 2018 (2, subgenotype B3). The additional cases were associated with alternative diagnoses other than AFM, including brainstem encephalitis, Guillain–Barré syndrome, myeloradiculitis, and spinal cord ischemia, which suggests that infection with EV-D68 can have a spectrum of severe neurologic complications, (*25*), a characteristic also seen with EV-A71 (*26*). However, without a positive CSF specimen, a positive stool specimen does not confirm causation in central nervous system viral infections. Virologic testing of stool specimens is the standard for poliomyelitis diagnosis, but this approach is problematic for EV-D68. EV-D68 shares biological characteristics, including temperature sensitivity, with genetically related rhinovirus species and grows preferentially in cell culture at 33°C, rather than 36°C, as was used by the NERL for enterovirus culture of stool specimens (*27*). EV-D68 is more likely to be detected in respiratory specimens than stool specimens (*28*), and collection of both of these types of specimens from cases of neurologic illness would improve rates of enterovirus typing (*29*). Worldwide, scientists acknowledge that the virus is increasingly detected, yet it is not known whether this increase represents an emerging pathogen or improved diagnostics (*30*).

From 2010 on, we observed peaks in AFM every 2–3 years. Southeast Asia typically observes a similar cyclical pattern of EV-A71 outbreaks. Authorities have assumed this pattern is a birth cohort effect (i.e., a periodic build-up of sufficiently large populations of susceptible children to sustain transmission) (*31*). More recently, models from China and the United States have found that climate, not demography, is a probable driver of enterovirus outbreaks; the average temperature and amount of water vapor in the air potentially explains variations in transmission (*32*,*33*). Subtyping of EV-A71 in Australia during 2010 and 2011 showed the dominant strain to be B5 (*12*). During 2013, a sudden increase in AFP cases associated with EV-A71 coincided with the introduction from China and Southeast Asia of the EV-A71 C4a strain, which was associated with more severe neurologic complications (*15*). EV-A71 cases in our study corresponded to 2 key outbreaks of EV-A71 that were described independently during our surveillance period (*15*,*34*). These studies support the contention that the AFP surveillance system in Australia can monitor NPEVs associated with AFM in the future.

Limitations of retrospective identification of AFM by using the AFP surveillance data include that underreporting might have occurred because of a failure of clinicians to diagnose or report AFP to APSU and PAEDS. However, pediatricians and PAEDS nurses were well-versed in reporting criteria, and clinical and laboratory data provided were usually sufficient to enable the PEP to confirm the AFP diagnosis and often the cause. Although the AFP surveillance questionnaire was standardized, the MRI findings were not reported in a standardized way. Specifically, because reports did not report enhancement of the spinal cord restricted to or predominately involving the gray matter, failure to classify AFP cases as AFM cases was plausible. Nevertheless, clinical manifestations of AFM showed similar demographic and clinical features with those for AFM described elsewhere. Lack of consistent stool samples, CSF collection, and respiratory specimens, limit our ability to exclude other pathogens. The low frequency of AFM cases might result in incidence estimates lacking precision and limited additional epidemiologic analyses.

Australia uses the systematic and continuous collection of AFP cases of the AFP surveillance system to ensure its capability to detect polio importations and maintain its polio-free status under WHO guidelines. The strengths of the surveillance system are its longstanding and centralized assessment of cases by an expert panel. The PEP includes pediatricians, neurologists, virologists, and surveillance nurses, and there is the ability to reach out to reporting pediatricians for additional patient information if required. Since 2018, there has been a concerted effort by the PEP to consider AFM alongside other neuroanatomical differential diagnoses of AFP in children, and AFM can now be captured as a final PEP classification.

Retrospective identification of AFM going back 20 years by using the AFP surveillance system is novel because many high-income countries have not maintained an AFP surveillance system that meets the WHO international surveillance targets. Furthermore, the system enables us to provide an estimate of AFM incidence in Australia, which was noted to be rare, but with a sustained occurrence from 2010 on. The AFP surveillance system is well-positioned to capture cases of AFM in children, and the centralized panel of experts who assess and classify each reported case of AFP are well-placed to monitor future trends in AFM in Australia.
